# Morphometric Analysis of Thigh Perforator Arteries Using CT Angiography: A Bilateral Comparative Study

**DOI:** 10.3390/jcm15208127

**Published:** 2026-10-20

**Authors:** Hurriyet Cetinok, Ayhan Comert

**Affiliations:** 1 Department of Anatomy, Faculty of Medicine, Istanbul Atlas University, 34403 Istanbul, Turkey; 2 Department of Anatomy, Faculty of Medicine, Ankara University, 06630 Ankara, Turkey; comertayhan@yahoo.com

**Keywords:** computed tomography angiography, perforator flap, anterolateral thigh, anteromedial thigh, posteromedial thigh, vascular anatomy

## Abstract

**Background/Objectives:** The thigh provides several perforator-based donor sites, but comparative computed tomography angiographic data describing anterolateral thigh (ALT), anteromedial thigh (AMT), and posteromedial thigh (PMT) perforators bilaterally remain limited. This study aimed to characterize CTA measurability, caliber, and side-to-side variability across these three territories. **Methods:** A total of 176 archived CTA records were screened. After predefined clinical/imaging exclusions and reconciliation of duplicated protocols, 49 unique patients with bilateral thigh imaging (98 lower limbs) were included. Patient-level measurable-perforator rates with 95% Wilson confidence intervals (CIs) were calculated. Side-to-side measurability was assessed with exact McNemar tests, territory differences with Cochran’s Q tests, and paired diameters with Wilcoxon signed-rank tests; Holm correction was applied within families of multiple comparisons. **Results:** PMT perforators were measurable in 75.5% of patients on each side (95% CI 61.9–85.4%), ALT in 51.0% on the left (37.5–64.4%) and 61.2% on the right (47.2–73.6%), and AMT in 24.5% on the left (14.6–38.1%) and 42.9% on the right (30.0–56.7%). Side-to-side measurability differed for AMT (exact McNemar *p* = 0.0117) but not ALT (*p* = 0.180) or PMT (*p* = 1.000). Paired ALT maximum diameter was 1.86 ± 0.27 mm on the left and 1.59 ± 0.42 mm on the right, but the bilateral difference was not statistically significant after Holm correction (adjusted *p* = 0.0588). No territory showed a statistically significant bilateral diameter difference after multiplicity correction, and overall maximum diameter did not differ among territories (*p* = 0.408). Among 17 patients with no measurable ALT perforator on either side, 15 (88.2%; 95% CI 65.7–96.7%) had a measurable AMT or PMT perforator. **Conclusions:** Archived CTA demonstrates substantial regional and bilateral variability in thigh perforator measurability. The three-territory comparison provides anatomical information that may inform individualized preoperative assessment, while the exploratory laterality findings require confirmation using standardized acquisition and reproducibility testing.

## 1. Introduction

The development of perforator flap surgery has enabled the transfer of skin and subcutaneous tissue while preserving the underlying musculature and reducing functional sacrifice at the donor site [1]. Among the available donor regions, the thigh is particularly versatile because it provides several anatomically distinct perforator-based options with favorable tissue characteristics and potential for primary donor-site closure. The anterolateral thigh (ALT) flap is the most established of these options and has become a workhorse flap for soft-tissue reconstruction [2,3]. However, the anteromedial thigh (AMT) and posteromedial thigh (PMT), including the profunda artery perforator (PAP) territory, provide additional reconstructive alternatives when ALT perforators are absent, small, anatomically unfavorable, or unsuitable for the intended flap design.

The ALT flap was originally described by Song et al. and is classically based on septocutaneous or musculocutaneous perforators arising predominantly from the descending branch of the lateral circumflex femoral artery (LCFA) [2]. Its long vascular pedicle, potentially large skin paddle, adaptable tissue volume, and broad clinical applicability have contributed to its widespread use [3]. Nevertheless, ALT perforator anatomy demonstrates considerable variation in vessel number, origin, caliber, location, and intramuscular course [4,5]. Smith et al., in a systematic review of ALT perforator variability, demonstrated substantial heterogeneity in perforator characteristics [4], while Rozen et al. similarly emphasized the variable anatomy encountered during flap harvest [5]. Such variability is clinically relevant because difficult intramuscular dissection, small-caliber vessels, or an unfavorable perforator position may complicate flap elevation and necessitate selection of an alternative perforator or donor territory.

The AMT perforator flap represents an important complementary option within the anterior thigh. Although less extensively characterized than the ALT flap, anatomical and clinical investigations have demonstrated that AMT perforators can provide suitable vessels for free tissue transfer [6,7,8,9]. Yu and Selber characterized clinically relevant AMT perforator patterns and recommended planning the AMT flap as a complement to the ALT flap rather than as the primary flap [6]. A cadaveric anatomical study by Hupkens et al. demonstrated that suitable AMT perforators may arise from all three main thigh vessels and that both septocutaneous and musculocutaneous patterns are encountered [7]. Anatomical studies have further characterized the vascular basis of the anteromedial thigh and reinforced its potential value in flap design [8]. Clinically, the AMT flap may therefore complement the ALT flap when lateral perforators are unsuitable or when a more medially positioned skin paddle is advantageous [9].

The posterior and posteromedial thigh constitute another distinct perforator donor territory. Perforators originating from the profunda femoris artery form the vascular basis of the PMT/PAP flap [10]. Cadaveric and CTA studies have demonstrated an anatomical basis for PAP flap harvest, including clinically useful pedicle length and vessel caliber [10]. The PAP flap has also been investigated for head and neck reconstruction, with detailed vascular characterization and successful clinical application reported [11]. Clinical series have further demonstrated the versatility of PAP flaps for oncological soft-tissue reconstruction [12]. The PAP flap has also been applied successfully in autologous breast reconstruction, further illustrating the versatility of this donor region [13]. Collectively, these findings support viewing the thigh not as a single ALT-centered donor site but as a multidimensional perforator donor complex containing complementary lateral, anteromedial, and posteromedial vascular territories.

This broader anatomical perspective is consistent with the perforasome concept, according to which individual perforators supply specific cutaneous vascular territories that can be strategically incorporated into flap design [14]. Accurate characterization of perforator distribution is therefore relevant to both flap selection and operative planning. Computed tomography angiography (CTA) provides a non-invasive means of evaluating perforator anatomy in vivo and allows assessment of vascular caliber, origin, course, and bilateral distribution before surgery. CTA studies have demonstrated clinically relevant anatomical variation within the ALT territory and have emphasized the potential value of preoperative vascular mapping [15]. Similar CTA-based investigations have characterized AMT perforators [16] and evaluated bilateral symmetry of ALT perforators [17]. CTA has also been used to define the anatomical characteristics of profunda artery perforators [18] and to further demonstrate the variability of ALT perforator caliber, origin, and course [19].

Despite these advances, previous CTA investigations have predominantly evaluated individual perforator territories rather than directly characterizing the ALT, AMT, and PMT regions bilaterally within the same imaging cohort [15,16,17,18,19]. Consequently, comparative information regarding the measurable frequency, caliber, and side-to-side variability of these three major thigh perforator territories remains limited. A more integrated assessment may be clinically useful when the preferred perforator is absent or suboptimal, and an alternative donor territory must be considered.

Therefore, the present study aimed to use CTA to comparatively characterize the morphometric features, measurable frequency, and bilateral variability of ALT, AMT, and PMT perforator arteries. By evaluating these territories within the same cohort, we sought to provide an integrated anatomical framework for comparing alternative thigh perforator territories; no surgical outcomes or flap-selection efficacy endpoints were assessed.

## 2. Materials and Methods

### 2.1. Study Design and Ethical Considerations

This retrospective observational study evaluated the computed tomography angiography (CTA) anatomy of thigh perforator arteries. Archived radiological examinations were obtained from the Clinical and Developmental Anatomy (C&DA) Department of Albert Einstein College of Medicine. 

The study was exempt from formal ethical approval because it involved retrospective analysis of previously acquired radiological data, in accordance with the exemption categories specified in Einstein IRB Citation 104(d). No additional imaging examination, intervention, or exposure to ionizing radiation was performed for the purposes of this investigation. The radiological and demographic data used for analysis were anonymized before assessment.

### 2.2. Study Population and Dataset Preparation

A total of 176 archived CTA records were initially screened for eligibility. Examinations were eligible when both thighs were adequately covered, and the arterial anatomy was sufficiently visualized to permit evaluation of perforator vessels within the ALT, AMT, and PMT territories. The examinations had been acquired for heterogeneous clinical indications, including oncologic assessment, vascular/ischemic symptoms, trauma or orthopedic conditions, and occasional flap-related evaluation. Of the 176 screened records, 124 were excluded because they met one or more predefined clinical or imaging exclusion criteria. The source screening record did not preserve a mutually exclusive count for each individual exclusion reason; accordingly, the reasons can be listed but cannot be itemized numerically without reconstructing the original screening process. The remaining 52 potentially eligible CTA records underwent protocol-level quality control. Review of protocol identifiers identified three duplicated records. Identical duplicated entries were counted only once, whereas complementary measurements associated with the same patient protocol were consolidated into a single patient-level record.

**Figure 1:**
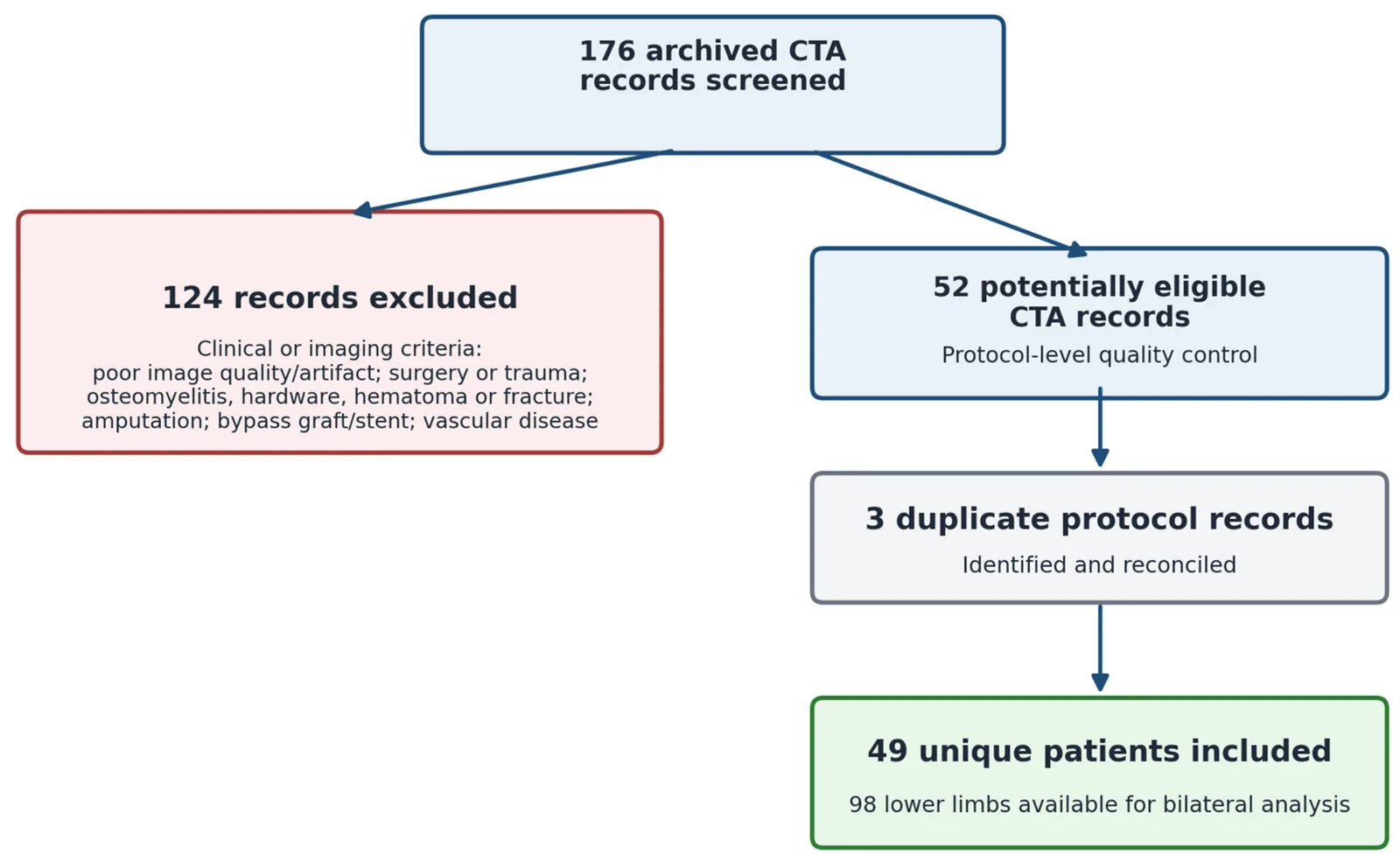
Flow diagram of CTA record screening, eligibility assessment, duplicate-protocol reconciliation, and selection of the final analytical cohort. CTA, computed tomography angiography.

Following screening and protocol-level quality control, the final study population comprised 49 unique patients and 98 lower limbs available for bilateral evaluation (Figure 1).

Examinations were excluded in cases of inadequate arterial opacification, poor image quality, incomplete bilateral thigh coverage, or substantial motion or metal-related artifacts preventing reliable perforator assessment. Additional exclusion criteria were previous thigh surgery or clinically significant trauma; osteomyelitis involving the evaluated lower extremity; orthopedic hardware affecting the evaluated region; thigh hematoma; femoral or periarticular fracture; major amputation involving the knee or thigh; lower-extremity arterial bypass grafting; vascular stent placement affecting the evaluated arterial system; severe peripheral arterial disease or other major vascular pathology; and diabetes mellitus accompanied by clinically significant peripheral vascular disease or vascular complications affecting the evaluated lower extremity. Individual territory-specific measurements were treated as missing when the relevant perforator could not be visualized with sufficient confidence or its diameter could not be reliably determined.

A non-measurable perforator was considered missing or non-measurable for morphometric analysis rather than being automatically classified as anatomically absent. The final cohort of 49 patients therefore represents an availability-based retrospective sample rather than a prospectively sampled population. Selection depended on bilateral coverage, adequate vascular visualization, and the absence of conditions that would preclude reliable assessment. No selection was made on the basis of measured perforator diameter, side-to-side difference, or a predetermined ALT/AMT/PMT pattern. Nevertheless, because eligibility required interpretable archived imaging, selection bias and spectrum bias cannot be excluded.

### 2.3. CTA Acquisition and Image Assessment

CTA examinations had originally been obtained for clinical purposes and were retrospectively accessed through the institutional Picture Archiving and Communication System (PACS). Multidetector CTA was performed during the arterial phase following intravenous administration of 140 mL of ISOVUE®-370 (iopamidol injection 76%; Bracco Diagnostics Inc., Monroe Township, NJ, USA) iodinated contrast medium. The clinical acquisition extended from the level of the renal arteries to the feet, whereas the anatomical region evaluated in the present study extended from the iliac crest to the distal femur. The examinations were obtained with the patient in a neutral anatomical position. CTA was acquired using a 64-detector-row multidetector CT system from GE Healthcare (Milwaukee, WI, USA). Thin-section axial images were reconstructed at 0.625 mm, with additional 2.5-mm reconstructed images available in the archived dataset. Post-processing included sagittal and coronal multiplanar reconstructions and axial and multiplanar maximum-intensity-projection (MIP) reconstructions using 10-mm and 20-mm slab thicknesses, together with three-dimensional volume-rendered (VR) images. Axial images, multiplanar reconstructions, MIP images, and VR reconstructions were reviewed in combination for vessel identification, anatomical interpretation, and morphometric assessment. Although these technical parameters could be recovered from the archived imaging records, examination-specific rotation time, reconstruction field of view, matrix, reconstruction kernel, and measurement-window settings were not uniformly recoverable for all examinations and were therefore not retrospectively inferred. The CTA measurements were initially performed by a radiologist and were subsequently reviewed and morphometrically evaluated by the first author, an anatomist experienced in radiological anatomy, under the supervision of the Head of the Department, who is a radiologist. Radiological consultation was available for vessel identification, anatomical interpretation, and measurement verification. This sequential review provided radiological oversight of the morphometric assessment; however, the original retrospective study was not designed as a blinded three-reader reproducibility study, and separate independent reader-level measurement files were not retained.

Perforator identification and assessment were conceptually based on previously described CTA evaluation of thigh perforators. The profunda femoris artery and its perforating branches were identified by tracing their course on sequential axial images and multiplanar reconstructions. The locations of identifiable perforators were subsequently marked on three-dimensional volume-rendered skin-surface reconstructions, including posterior views, to demonstrate their relationships to the cutaneous surface.

Both thighs were assessed separately to permit bilateral morphometric comparison.

Because this was a retrospective investigation using archived clinical CTA examinations, no additional image acquisition, contrast administration, dedicated research imaging protocol, or exposure to ionizing radiation was performed for this study.

### 2.4. Identification and Morphometric Assessment of Perforators

Perforator arteries were identified by following vascular branches on sequential CTA images and multiplanar reconstructions toward the fascial and subcutaneous tissues of the thigh. Territory assignment was topographic: ALT denoted perforators coursing to the anterolateral thigh, AMT denoted perforators coursing to the anteromedial thigh, and PMT denoted perforators coursing to the posteromedial thigh. In this manuscript, PMT refers to the posteromedial cutaneous territory, whereas PAP refers specifically to a profunda femoris artery perforator that may supply this territory; the terms are therefore related but not interchangeable.

For each perforator that could be confidently traced and measured on the archived CTA, the recorded vessel diameter was retained in millimeters and classified by territory and side. A fixed numerical measurability threshold had not been prospectively defined in the original retrospective measurement protocol; the smallest recorded measurable diameter in the dataset was 0.70 mm. Likewise, the exact vessel level at which the diameter was recorded (for example, origin versus fascial emergence) was not encoded uniformly in the finalized analytic worksheet. Consequently, the diameter data should be interpreted as retrospective recorded CTA measurements rather than standardized measurements at a single prespecified vessel level. This limitation precludes direct one-to-one comparison with studies that report caliber exclusively at the source-vessel origin or at the deep fascia.

When more than one measurable perforator was present within the same anatomical territory and side, the individual vessel diameters were retained for vessel-level descriptive analysis. For patient-level bilateral statistical comparisons, the largest measurable perforator diameter within each territory and side was selected to provide one representative measurement per territory per thigh and to avoid treating multiple vessels from the same thigh as independent observations.

The presence of at least one reliably measurable perforator within a defined territory was recorded at the patient level. Accordingly, the frequency reported in this study represents the CTA measurable-perforator rate rather than the absolute anatomical prevalence of perforators.

Where arterial origin was identifiable and documented, the recorded vascular origin was additionally retained for descriptive analysis. When more than one origin was recorded for the same protocol, individual origin labels were retained while duplicate entries from repeated protocol records were counted only once.

**Figure 2:**
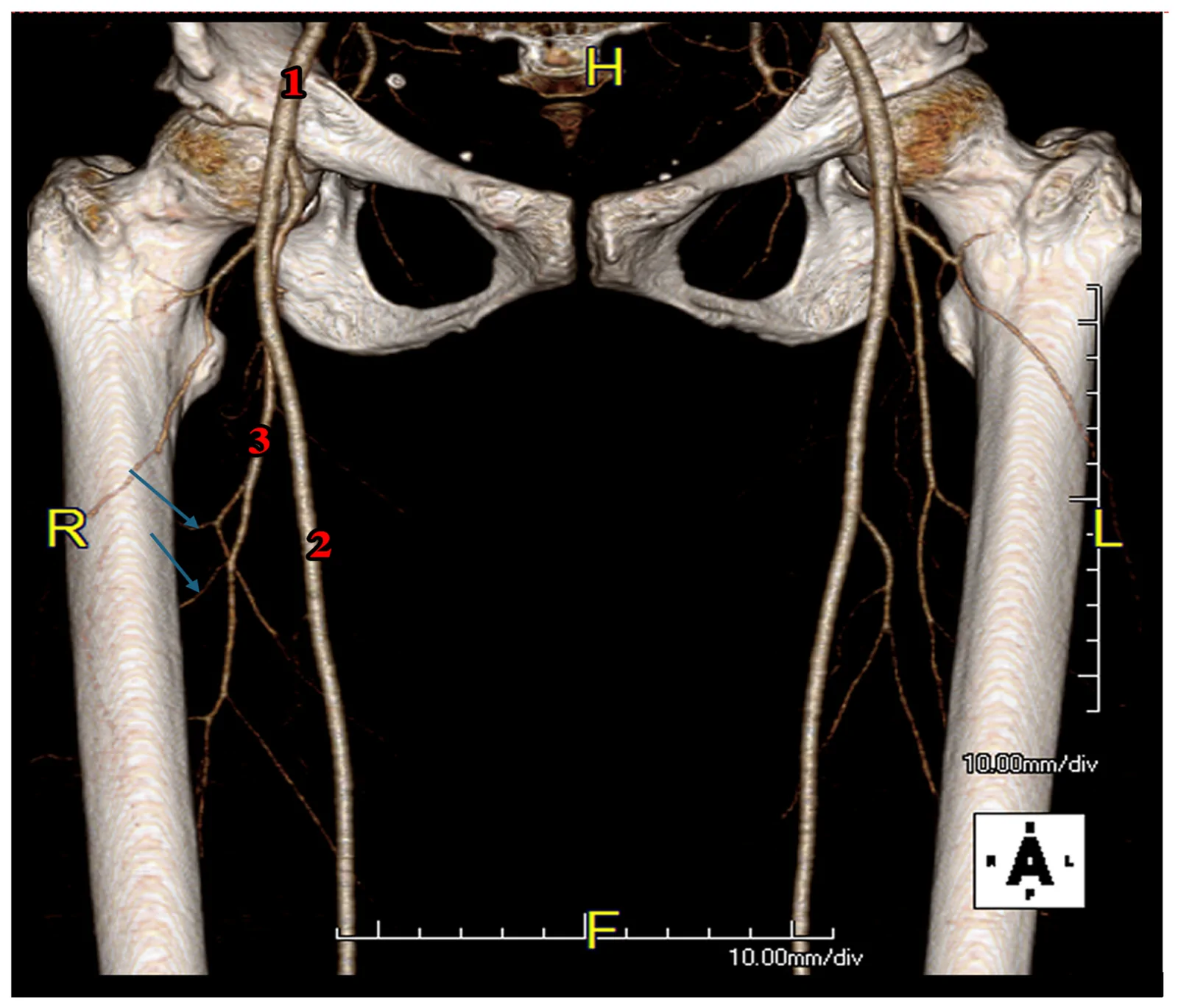
Anterior maximum-intensity-projection (MIP) reconstruction of bilateral CT angiography demonstrating anteromedial thigh perforators (blue arrows). The blue arrows identify the selected AMT perforating branches in relation to the larger arterial landmarks: 1, common femoral artery (CFA); 2, superficial femoral artery (SFA); 3, profunda femoris artery (PFA). R, right; L, left; H, head (cranial); F, foot (caudal).

**Figure 3:**
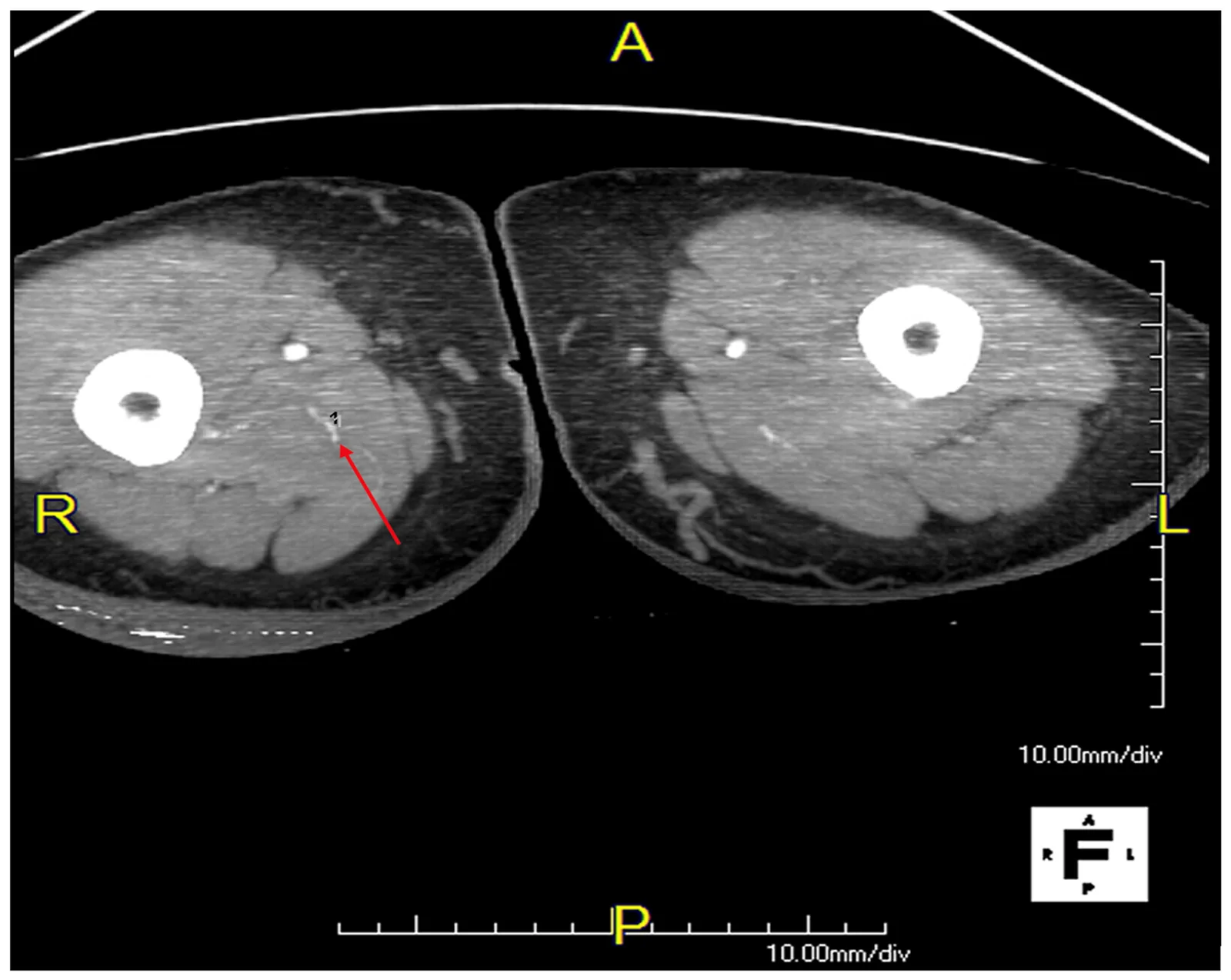
Axial CT angiographic image demonstrating a right posteromedial thigh perforator arising from the right profunda femoris artery (RPFA) and coursing toward the fascia between the adductor magnus and gracilis muscles (red arrow). A 3.0 mm diameter was recorded for this example in the source dataset; because the measurement level was not uniformly encoded across the retrospective dataset, the figure should not be interpreted as defining a standardized measurement level for all cases. R, right; L, left; A, anterior; P, posterior.

**Figure 4:**
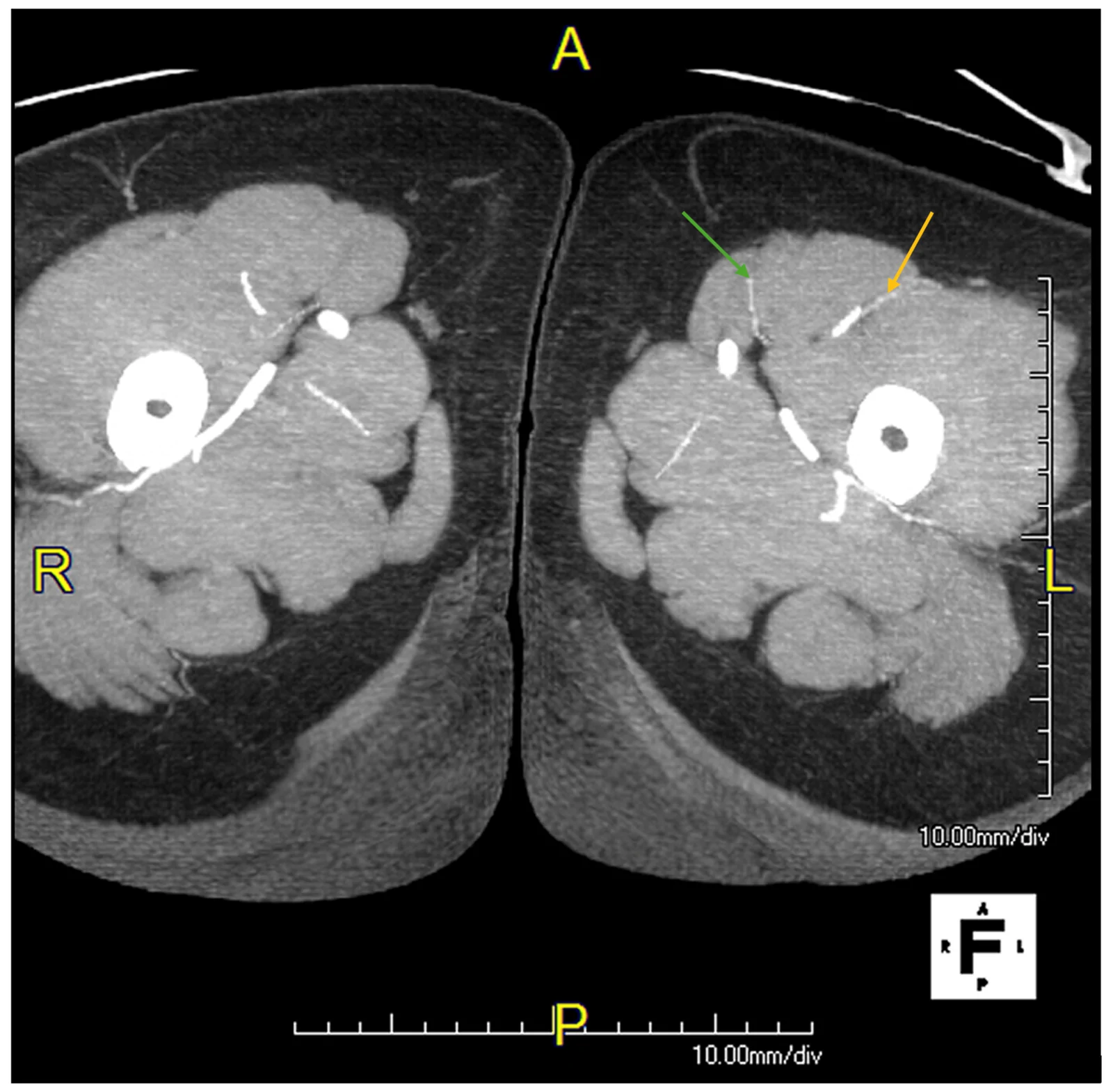
Axial CT angiographic image demonstrating simultaneous visualization of left thigh perforators. The green arrow indicates the anteromedial thigh (AMT) perforator, and the orange arrow indicates the anterolateral thigh (ALT) perforator. R, right; L, left; A, anterior; P, posterior.

Representative CTA reconstructions illustrating perforator identification, fascial penetration, and simultaneous visualization of adjacent perforator territories are presented in Figure 2, Figure 3 and Figure 4.

### 2.5. Data Quality Control and Handling of Missing Values

Before statistical analysis, the complete dataset was reviewed for duplicate protocol numbers, missing observations, non-numeric entries, and cells containing measurements of more than one perforator.

Decimal notation was standardized before analysis. When multiple numeric measurements were recorded within a single anatomical territory, these were interpreted as separate measurable perforators for vessel-level descriptive analysis.

Entries in which vessel diameter could not be reliably determined were treated as missing values and excluded from diameter-based calculations. No numerical imputation was performed.

Duplicate protocol records were consolidated before all patient-level analyses. Consequently, patient-level frequencies and inferential statistical comparisons were based on 49 unique patients rather than the original 52 spreadsheet records.

Although the measurements underwent sequential radiological assessment, anatomical morphometric review, and senior radiological supervision, the archived retrospective dataset was not designed as a formal blinded multi-reader reproducibility study. Separate independent reader-level measurements and repeated-reading data were not retained in the finalized analytic file. Accordingly, formal interobserver reliability statistics (such as ICC for continuous measurements or kappa for categorical measurements) and intraobserver ICC could not be calculated retrospectively from the finalized dataset without undertaking a re-reading of the original DICOM examinations. The absence of prospectively planned formal reproducibility testing is therefore treated as a methodological limitation, and the laterality findings are interpreted as exploratory and requiring confirmation.

### 2.6. Statistical Analysis

Statistical analyses were based on 49 unique patient protocols after duplicate reconciliation. Continuous variables were summarized as mean ± standard deviation (SD), median, and minimum–maximum values, as appropriate, and categorical variables as frequencies and percentages. For each measurable-perforator proportion, 95% Wilson confidence intervals were calculated.

Measurable-perforator rates were calculated separately for each anatomical territory and side as the proportion of unique patients in whom at least one perforator could be reliably measured on CTA. Left–right differences in this paired binary endpoint were assessed using exact McNemar tests. Differences in measurability among ALT, AMT, and PMT within the same patients were assessed using Cochran’s Q test, followed by pairwise exact McNemar comparisons with Holm adjustment.

For diameter analyses, left and right measurements from the same patient were treated as paired observations. The largest measurable perforator within each territory and side was used as the patient-level representative value. To ensure consistent reporting across territories, all three primary paired diameter comparisons used the same two-sided asymptotic Wilcoxon signed-rank procedure. Hodges–Lehmann paired location-shift estimates with bootstrap 95% confidence intervals and rank-based effect-size estimates (|r|) were calculated as measures of precision and effect magnitude. The three ALT/AMT/PMT side-to-side diameter tests were considered one family and were additionally adjusted using the Holm method.

Comparisons among ALT, AMT, and PMT maximum measurable diameters were performed using the Friedman test in patients with complete measurements for all territories being compared. A sensitivity analysis was also performed for ALT laterality in all 32 patients with at least one measurable ALT side by imputing a non-measurable side at 0.50 mm and, separately, at 0.70 mm; this analysis was designed to examine the alternative assumption that non-measurability reflected a vessel below the measurement range rather than image quality.

Vessel-level descriptive analyses and patient-level inferential analyses were deliberately separated to minimize the risk of pseudoreplication associated with multiple perforators originating from the same patient.

All tests were two-sided. Unadjusted *p*-values are shown where they describe the prespecified paired comparisons, with Holm-adjusted values reported for the corresponding multiple-comparison family. Statistical significance after multiplicity correction was defined as adjusted *p* < 0.05.

### 2.7. Generative AI Use

During preparation of this manuscript, ChatGPT (GPT-5.6, OpenAI, San Francisco, CA, USA; https://chatgpt.com/; accessed on 23 August 2026) was used to assist with language editing, structural organization, and preparation of graphical presentations based on author-provided data. All scientific content, interpretations, citations, and final manuscript text were reviewed and approved by the authors, who take full responsibility for the published content.

## 3. Results

### 3.1. Study Cohort and Data Quality Control

A total of 176 archived CTA records were screened. Of these, 124 were excluded according to the predefined clinical and imaging eligibility criteria. The remaining 52 records underwent protocol-level quality control, during which three duplicated protocol numbers were identified and reconciled. Consequently, the final analysis included 49 unique patients and 98 lower limbs (Figure 1). The cohort comprised 28 women (57.1%), 20 men (40.8%), and one patient (2.0%) for whom sex was not recorded. Mean age was 53.5 ± 13.6 years (range, 17–79 years). Clinical indications for the included examinations were heterogeneous and included oncologic, vascular/ischemic, trauma/orthopedic, and flap-related evaluations. BMI and other anthropometric covariates were not available in the analytic dataset and therefore could not be evaluated as potential determinants of vessel caliber. No numerical imputation was performed for the primary complete-case diameter analysis.

### 3.2. Patient-Level Measurable-Perforator Rates

PMT perforators were measurable in 37 of 49 patients (75.5%; 95% CI 61.9–85.4%) on both the left and right sides. ALT perforators were measurable in 25 patients on the left (51.0%; 95% CI 37.5–64.4%) and 30 on the right (61.2%; 95% CI 47.2–73.6%), whereas AMT perforators were measurable in 12 patients on the left (24.5%; 95% CI 14.6–38.1%) and 21 on the right (42.9%; 95% CI 30.0–56.7%). Exact paired McNemar testing showed no left–right measurability difference for ALT (*p* = 0.180) or PMT (*p* = 1.000), but AMT measurability was higher on the right (*p* = 0.0117). Across the three territories, measurability differed on both the left (Cochran Q = 24.68, *p* < 0.001) and right (Q = 11.03, *p* = 0.004). After Holm-adjusted pairwise testing, all three left-sided territory comparisons remained significant (ALT vs. AMT adjusted *p* = 0.021; ALT vs. PMT adjusted *p* = 0.021; AMT vs. PMT adjusted *p* < 0.001), whereas on the right only AMT vs. PMT remained significant (adjusted *p* = 0.016). Considering either side, at least one measurable PMT perforator was identified in 41 patients (83.7%), compared with 32 (65.3%) for ALT and 22 (44.9%) for AMT. Bilateral measurable perforators were present in 33 patients (67.3%) for PMT, 23 (46.9%) for ALT, and 11 (22.4%) for AMT.

**Figure 5:**
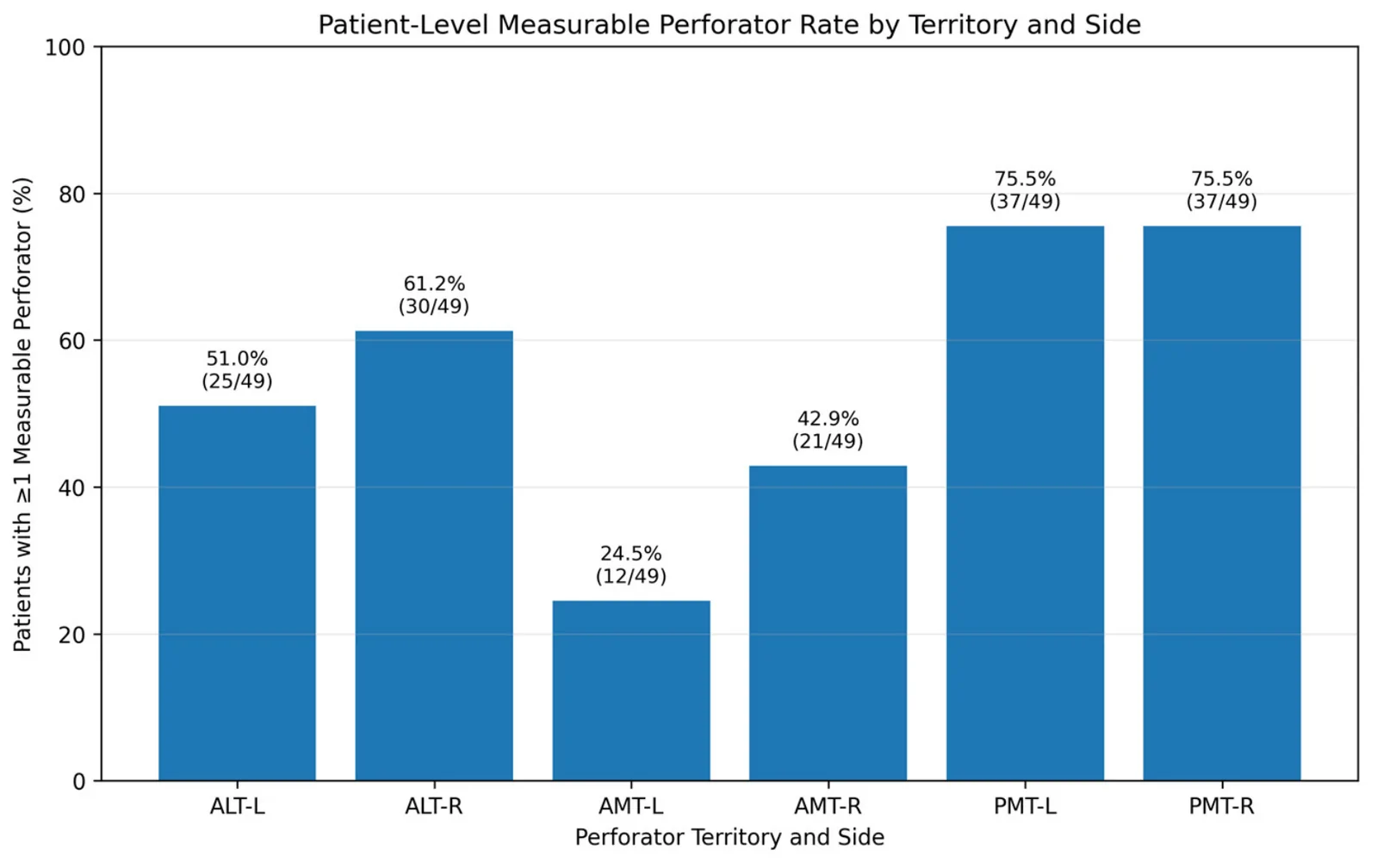
Patient-level measurable-perforator rates by anatomical territory and side. Percentages are based on 49 unique patients after duplicate-protocol consolidation.

The integrated three-territory analysis provided clinically relevant conditional information that cannot be obtained from a single-territory series. Seventeen patients had no measurable ALT perforator on either side; of these, 15 (88.2%; 95% CI 65.7–96.7%) had at least one measurable AMT or PMT perforator. At least one recorded perforator measuring ≥1.5 mm on either side was present in 25/49 patients for ALT (51.0%; 95% CI 37.5–64.4%), 17/49 for AMT (34.7%; 95% CI 22.9–48.7%), and 37/49 for PMT (75.5%; 95% CI 61.9–85.4%). These values are imaging-based descriptive findings and should not be interpreted as operative suitability or flap success (Figure 5).

### 3.3. Perforator Diameter and Bilateral Morphometric Comparison

**Figure 6:**
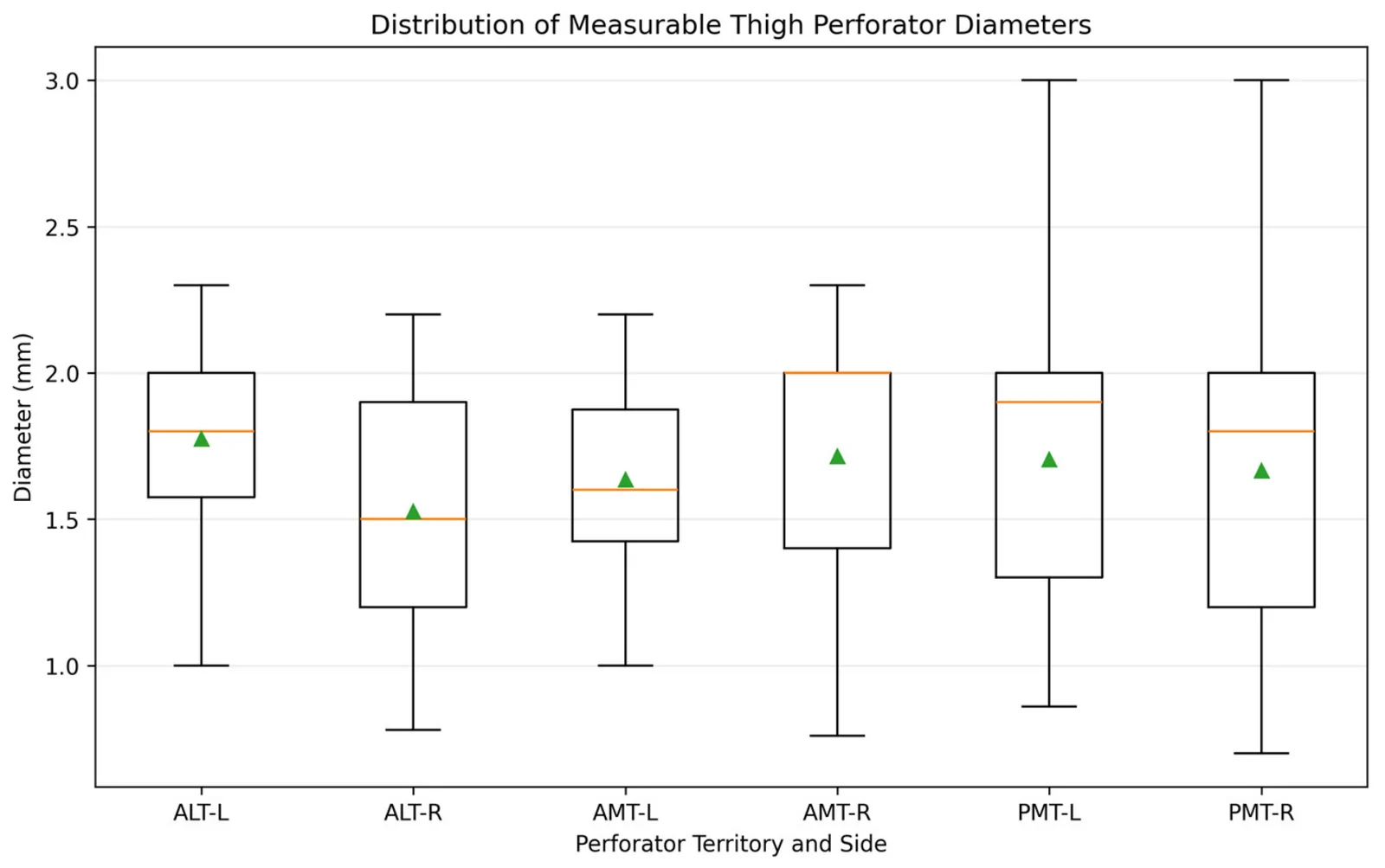
Vessel-level distribution of measurable perforator diameters in the ALT, AMT, and PMT territories bilaterally. Boxes represent the interquartile range, horizontal lines the median, triangles the mean, and whiskers the observed range. This figure is descriptive; inferential side-to-side comparisons were performed at the patient level as shown in Table 2.

Across all individually recorded measurable vessels, mean diameters ranged from 1.53 ± 0.41 mm for ALT-R perforators to 1.78 ± 0.34 mm for ALT-L perforators. AMT diameters measured 1.64 ± 0.32 mm on the left and 1.71 ± 0.41 mm on the right. PMT perforators demonstrated the broadest diameter range, extending to 3.00 mm bilaterally, with mean diameters of 1.70 ± 0.50 mm on the left and 1.67 ± 0.54 mm on the right (Table 1 and Figure 6).

For inferential left–right comparisons, one representative value per territory and side was defined as the largest measurable perforator diameter in each patient. Paired ALT measurements were available in 23 patients; the left-sided maximum was 1.86 ± 0.27 mm and the right-sided maximum was 1.59 ± 0.42 mm (unadjusted asymptotic Wilcoxon *p* = 0.0196; Hodges–Lehmann shift +0.30 mm, bootstrap 95% CI 0.00 to +0.50 mm; |r| = 0.55). After Holm correction for the three paired territory comparisons, the ALT result no longer met the adjusted significance threshold (adjusted *p* = 0.0588). For AMT (n = 11), the corresponding values were 1.68 ± 0.30 versus 1.72 ± 0.45 mm (*p* = 0.715; Hodges–Lehmann 0.00 mm, 95% CI −0.30 to +0.10 mm; |r| = 0.18; adjusted *p* = 1.000). For PMT (n = 33), they were 1.81 ± 0.47 versus 1.81 ± 0.52 mm (*p* = 0.773; Hodges–Lehmann 0.00 mm, 95% CI −0.25 to +0.25 mm; |r| = 0.06; adjusted *p* = 1.000).

Sensitivity analysis of ALT laterality included all 32 patients with at least one measurable ALT side. When a non-measurable side was imputed as 0.50 mm, the left–right comparison was not significant (*p* = 0.564); using 0.70 mm, the comparison was likewise not significant (*p* = 0.380). Thus, the nominal complete-case ALT laterality signal is sensitive to assumptions about why a contralateral perforator was non-measurable and should be interpreted cautiously.

**Table 2:** Patient-level paired comparison of maximum measurable perforator diameter between sides. A. Precision, effect size, and multiplicity for paired maximum-diameter comparisons.

Territory	Paired *n*	Left Mean ± SD (mm)	Right Mean ± SD (mm)	Mean Difference L–R (mm)	Wilcoxon *p*-Value
ALT	23	1.86 ± 0.27	1.59 ± 0.42	+0.27	0.0196
AMT	11	1.68 ± 0.30	1.72 ± 0.45	−0.04	0.715
PMT	33	1.81 ± 0.47	1.81 ± 0.52	0.00	0.773
A					
**Territory**	**Paired *n***	**HL Shift, mm**	**Bootstrap 95% CI**	**|r|**	**Holm-Adjusted *p***
ALT	23	+0.30	0.00 to +0.50	0.55	0.0588
AMT	11	0.00	−0.30 to +0.10	0.18	1.000
PMT	33	0.00	−0.25 to +0.25	0.06	1.000

Unadjusted asymptotic Wilcoxon *p*-values are shown. Holm-adjusted *p*-values for the three-territory comparison family were 0.0588 (ALT), 1.000 (AMT), and 1.000 (PMT). Paired analyses used the largest measurable perforator diameter per territory and side for each patient.

### 3.4. Comparison Among Perforator Territories and Multiplicity

For the comparison among perforator territories, 16 patients had measurable ALT, AMT, and PMT values after patient-level aggregation. The Friedman test demonstrated no statistically significant difference in maximum measurable diameter among the three perforator territories (χ^2^ = 1.79, *p* = 0.408).

Multiple measurable perforators within the same territory and side were encountered across all regions. The highest frequency of multiple recorded perforators occurred in PMT-R (8/49, 16.3%), followed by ALT-R and PMT-L (6/49 each, 12.2%), AMT-R (4/49, 8.2%), ALT-L (3/49, 6.1%), and AMT-L (2/49, 4.1%). These observations further illustrate the within-territory anatomical variability of the thigh perforator network.

### 3.5. Recorded Vascular-Origin Distribution

**Figure 7:**
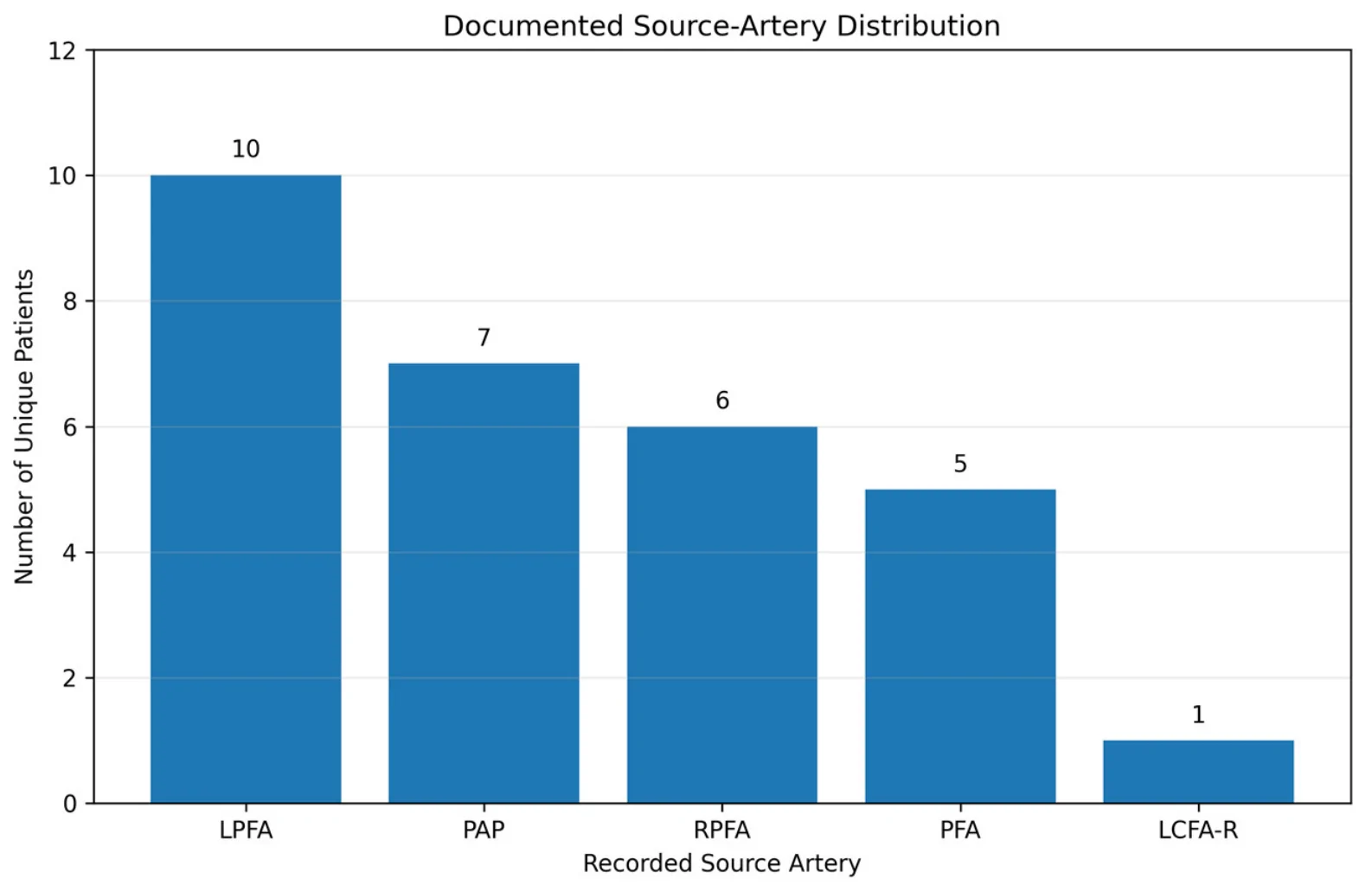
Distribution of documented vascular-origin labels after consolidation to unique patient protocols. Multiple origin labels could be recorded in the same patient; therefore, counts are not mutually exclusive. LPFA, left profunda femoris artery; RPFA, right profunda femoris artery; PFA, profunda femoris artery; PAP, profunda artery perforator; LCFA-R, right lateral circumflex femoral artery.

Vascular-origin information was documented in 21 of 49 unique patients (42.9%). Because more than one origin label could be recorded for an individual patient, the following frequencies are non-mutually exclusive. The left profunda femoris artery (LPFA) was documented in 10 patients (47.6% of patients with recorded origin data), profunda artery perforator (PAP) in 7 (33.3%), right profunda femoris artery (RPFA) in 6 (28.6%), profunda femoris artery without side specification (PFA) in 5 (23.8%), and right lateral circumflex femoral artery (LCFA-R) in 1 (4.8%) (Figure 7).

## 4. Discussion

The principal finding of the present CTA study is that the three major thigh perforator territories demonstrate distinct patterns of measurability and bilateral variability despite broadly comparable maximum vessel calibers. PMT was the most consistently measurable territory, whereas AMT demonstrated a significant side-to-side difference in the measurable-perforator rate. The complete-case ALT diameter comparison yielded an unadjusted *p* = 0.0196, but this did not remain significant after Holm correction (adjusted *p* = 0.0588) and was not robust in threshold-imputation sensitivity analyses. Accordingly, the ALT laterality observation should be viewed as exploratory rather than as evidence of consistent left-sided dominance. No significant overall difference in maximum measurable diameter was identified among the three territories (*p* = 0.408). Most importantly, among patients with no measurable ALT on either side, 88.2% had a measurable AMT or PMT perforator, illustrating the value of examining the thigh as a set of complementary territories within the same patient.

The ALT flap remains the most extensively characterized thigh-based perforator flap and has long been considered a reliable workhorse for soft-tissue reconstruction [3]. Nevertheless, its vascular anatomy is known to be heterogeneous. Systematic and anatomical investigations have demonstrated considerable variability in the number, origin, position, caliber, and intramuscular course of ALT perforators [4,5]. CTA studies have further confirmed this variability and demonstrated the value of preoperative mapping in identifying potentially favorable perforators and selecting the donor thigh [15,19]. In the present study, the mean vessel-level ALT diameter was 1.78 ± 0.34 mm on the left and 1.53 ± 0.41 mm on the right. When the largest measurable perforator in each patient was used for paired analysis, the left-sided maximum was numerically greater than the right-sided maximum, but the difference did not remain statistically significant after Holm correction (adjusted *p* = 0.0588).

The observed ALT laterality pattern is relevant when considered alongside the CTA study of Lu et al. [17], which demonstrated side-to-side variability in ALT perforator anatomy. However, our complete-case diameter result is sensitive to multiplicity correction and to assumptions regarding non-measurable contralateral vessels. It therefore should not be interpreted as evidence of universal left-sided dominance. Instead, the present data support the broader conclusion that contralateral ALT anatomy cannot automatically be assumed to be equivalent and that standardized prospective imaging is needed to quantify the magnitude and reproducibility of side differences.

The AMT territory demonstrated a different pattern. AMT perforators were measurable less frequently than ALT or PMT perforators in the present CTA cohort, particularly on the left side. Previous anatomical and clinical studies have likewise emphasized the variable nature of AMT perforators. Yu and Selber identified no AMT perforator in 21 of 100 thighs and demonstrated variability in both source vessel and perforator pattern [6]. Hupkens et al., in a cadaveric anatomical study, showed that suitable AMT perforators could arise from all three main thigh vessels and that both septocutaneous and musculocutaneous perforator patterns were present [7]. Visconti et al. used CTA to characterize AMT perforator emergence, caliber, length, course, and source vessel, further demonstrating that the AMT region contains a clinically relevant but variable perforator network [16].

The lower AMT measurable rates observed in the present study should not be directly interpreted as anatomical absence. Our endpoint was the presence of a perforator that could be confidently identified and measured on archived clinical CTA examinations, whereas previous surgical, cadaveric, and dedicated imaging investigations used different inclusion thresholds and anatomical definitions [6,7,16]. A fixed numeric CTA measurability threshold was not specified prospectively in our original retrospective protocol; the smallest recorded measurable diameter was 0.70 mm. Consequently, the present percentages represent CTA measurable-perforator rates rather than true anatomical prevalence. Notably, paired measurability was significantly greater on the right for AMT (*p* = 0.0117), whereas the paired diameter comparison among measurable AMT vessels was not significant (*p* = 0.715).

The PMT territory produced one of the most notable observations of the present analysis. PMT perforators were measurable in 75.5% of patients on each side, and at least one PMT perforator was measurable on either side in 83.7% of patients. PMT vessels also demonstrated the broadest observed diameter range, reaching 3.0 mm bilaterally. Saad et al. combined cadaveric dissection with CTA to establish the anatomical basis of the profunda femoris artery perforator flap and reported clinically useful pedicle length and vessel caliber [10]. Liu et al. further characterized PAP vascular anatomy and described its application in head and neck reconstruction [11]. Ito et al. reported PAP flap use across a range of oncological reconstructions, including head and neck defects, supporting the versatility of this donor territory [12], while Atzeni et al. reported favorable outcomes with PAP flaps in autologous breast reconstruction [13].

CTA has also contributed to characterization of the profunda artery perforator system. Sonda et al. used CTA to characterize the radiological architecture of posterior thigh perforators, including perforator number, caliber at multiple levels, and spatial relationships relevant to PAP flap planning [18]. Our finding that PMT perforators were the most consistently measurable vessels among the three territories therefore complements the existing anatomical and CTA literature. However, direct comparison of vessel diameters among studies should be undertaken cautiously because measurement location, vessel-selection criteria, imaging resolution, and definitions of a clinically relevant perforator differ substantially between investigations [10,11,18]. The present data support the PMT/PAP region as a substantial vascular territory but do not establish superiority of the PMT flap over ALT or AMT flaps.

This point is reinforced by the absence of a significant overall difference in maximum measurable diameter among ALT, AMT, and PMT perforators (*p* = 0.408). Although the territories differed in measurable frequency and variability, none demonstrated a statistically larger caliber overall. Therefore, perforator diameter alone is unlikely to provide a sufficient basis for donor-site selection. Flap planning must additionally consider perforator course, pedicle anatomy, cutaneous territory, required tissue volume, donor-site characteristics, operative positioning, and the reconstructive requirements of the recipient site [3,7,11,12,13,14]. This interpretation is consistent with the perforasome concept, which emphasizes that individual perforators support distinct but interconnected vascular territories and that flap design should account for the spatial characteristics of the selected vascular supply [14].

From a practical microsurgical perspective, the recorded mean calibers of approximately 1.5–1.8 mm fall within a range commonly encountered in small-vessel anastomosis, and PMT measurements reached 3.0 mm. In the present cohort, a perforator measuring at least 1.5 mm on either side was recorded in 51.0% of patients for ALT, 34.7% for AMT, and 75.5% for PMT. These imaging measurements should not be equated with operative suitability, because anastomotic feasibility also depends on vessel quality, pedicle length, recipient-vessel match, course, and the tissue requirements of the reconstruction.

Multiple measurable perforators were also observed within individual territories. The highest frequency of multiple perforators was recorded in the right PMT region, followed by the right ALT and left PMT regions. Although these findings are descriptive and should not be used to establish a hierarchy among donor sites, they further demonstrate that thigh perforator anatomy is not adequately represented by a single-vessel model. The presence of multiple potential perforators within a territory may provide flexibility in vessel selection, but this possibility should be confirmed prospectively and correlated with operative anatomy.

Vascular-origin data were available in only 21 of the 49 patients and therefore required particularly cautious interpretation. Profunda-related entries predominated among the documented origin labels, which is broadly compatible with established descriptions of the PMT/PAP territory [10,11,18]. However, because origin information was incomplete, more than one origin could be recorded in the same patient, and the available origin variable was not uniformly documented for every perforator territory, these findings should be considered descriptive rather than definitive. A larger prospectively collected CTA series with systematic vessel tracing would be required for a detailed comparison of source-vessel patterns.

Previous CTA investigations have demonstrated clinically relevant variation in ALT perforator origin, location, caliber, and course and have emphasized the potential value of preoperative mapping [15,19]. Dedicated CTA studies have similarly characterized AMT perforators [16], bilateral ALT anatomy [17], and PAP anatomy [18]. The present study adds a within-cohort comparison of three thigh territories. When a preferred territory contains a non-measurable perforator on archived CTA, assessment of the contralateral thigh or an alternative ALT, AMT, or PMT territory can provide additional anatomical information; however, the present study did not test whether this strategy improves surgical outcomes.

Clinical evidence from other cohorts has evaluated CTA-guided planning. Liu et al. retrospectively compared 32 patients undergoing ALT flap reconstruction of through-and-through cheek defects using handheld Doppler or preoperative CTA guidance [20]. Their results suggest that detailed preoperative imaging can influence donor-side planning in a specific clinical setting. The present study did not compare imaging strategies and did not evaluate operative outcomes. Because this investigation was a retrospective morphometric analysis of archived CTA data rather than a prospective surgical-validation cohort, paired intraoperative findings were not systematically available for comparison with the CTA measurements. Therefore, our data should be interpreted as comparative anatomical information rather than evidence that CTA itself improves flap selection, operative time, complication rates, or reconstructive outcomes.

Several limitations should be considered. First, this was an availability-based retrospective cohort drawn from archived clinical examinations obtained for heterogeneous indications; selection bias and spectrum bias cannot be excluded. Second, thin-section 0.625-mm images and additional 2.5-mm reconstructions, together with 10-mm and 20-mm MIP slab reconstructions and three-dimensional volume-rendered images, were recoverable from the archived imaging records. However, examination-specific rotation time, reconstruction field of view, matrix, reconstruction kernel, and measurement-window settings were not uniformly recoverable. Because the vessels measured were small, residual technical variation may be of the same order as some observed between-side differences. Third, a fixed prospective measurability threshold and a uniform vessel level for diameter measurement were not encoded in the original dataset; this limits reproducibility and cross-study comparison. Fourth, although the measurement workflow included radiological assessment, subsequent anatomical morphometric review, and senior radiological supervision, these steps did not constitute three independent blinded readings, and separate reader-level measurement files were not retained. Formal interobserver and intraobserver reliability statistics therefore could not be calculated from the finalized retrospective dataset. Fifth, complete paired sample sizes differed across territories, particularly for AMT, reducing precision; the confidence intervals and effect-size estimates should therefore be considered alongside *p*-values. Sixth, the nominal ALT complete-case laterality result did not remain significant after Holm correction and was not robust to threshold-imputation sensitivity analyses. Seventh, BMI and other anthropometric covariates were not available; therefore, obesity-related variation in perforator caliber could not be evaluated, and residual confounding related to body habitus cannot be excluded. The original screening log did not retain mutually exclusive counts for each of the 124 exclusion reasons. Eighth, non-measurable perforators were not assumed to be anatomically absent. Ninth, vascular-origin information was incomplete. In addition, because this was a retrospective imaging data collection and morphometric study rather than a prospective surgical-validation cohort, corresponding operative findings were not systematically available and the CTA findings could not be validated intraoperatively. Finally, pedicle length, standardized course type, and standardized surface-coordinate data were not available for complete comparative analysis. These variables are directly relevant to operative planning; their absence limits translation of the CTA morphometric findings to flap design and surgical selection. These limitations require cautious interpretation and prospective confirmation.

Despite these limitations, the study has several strengths. Bilateral CTA permitted evaluation of both thighs within the same individuals, thereby allowing true paired assessment of side-to-side differences. ALT, AMT, and PMT territories were examined within a single cohort using a consistent analytical framework, and vessel-level descriptive analyses were separated from patient-level inferential analyses to reduce the risk of pseudoreplication. In addition, duplicate protocol records were identified and consolidated before final analysis. These features provide an integrated view of thigh perforator morphology and highlight anatomical relationships that may be overlooked when each flap territory is considered independently.

## 5. Conclusions

CTA demonstrated distinct patterns of measurable-perforator frequency and bilateral variability among ALT, AMT, and PMT territories. PMT was the most consistently measurable territory, while AMT showed a significant side-to-side difference in measurability. The unadjusted ALT maximum-diameter laterality signal did not remain significant after Holm correction and was sensitive to assumptions regarding non-measurable contralateral vessels. Maximum measurable diameter did not differ significantly among the three territories overall.

The within-patient three-territory analysis indicates that alternative AMT or PMT perforators are frequently measurable when ALT is not measurable on either side, supporting the anatomical concept of complementary thigh perforator territories. These findings may inform preoperative anatomical assessment but do not demonstrate superiority of any flap, improvement in donor-site selection, or better surgical outcomes. Prospective studies using standardized CTA acquisition, prespecified measurement levels, reproducibility testing, anthropometric covariates, and intraoperative validation are warranted.

## Figures and Tables

**Table 1:** Precision of patient-level measurable-perforator rates and exact paired side comparison. A. Patient-level measurable-perforator rates and vessel-level morphometric characteristics.

Territory	Left Measurable *n* (%)	95% CI	Right Measurable *n* (%)	95% CI	Exact McNemar *p*
ALT	25 (51.0)	37.5–64.4	30 (61.2)	47.2–73.6	0.180
AMT	12 (24.5)	14.6–38.1	21 (42.9)	30.0–56.7	0.0117
PMT	37 (75.5)	61.9–85.4	37 (75.5)	61.9–85.4	1.000
A					
**Territory-Side**	**Measurable Patients, *n* (%)**	**Measured Vessels, *n***	**Mean ± SD (mm)**	**Median (mm)**	**Range (mm)**
ALT-L	25 (51.0)	28	1.78 ± 0.34	1.80	1.00–2.30
ALT-R	30 (61.2)	38	1.53 ± 0.41	1.50	0.78–2.20
AMT-L	12 (24.5)	14	1.64 ± 0.32	1.60	1.00–2.20
AMT-R	21 (42.9)	25	1.71 ± 0.41	2.00	0.76–2.30
PMT-L	37 (75.5)	43	1.70 ± 0.50	1.90	0.86–3.00
PMT-R	37 (75.5)	45	1.67 ± 0.54	1.80	0.70–3.00

ALT, anterolateral thigh; AMT, anteromedial thigh; PMT, posteromedial thigh; L, left; R, right; SD, standard deviation. Measurable-perforator rates use 49 unique patients as the denominator. Diameter statistics are vessel-level descriptive values and include all individually recorded measurable perforators after duplicate-protocol consolidation.

## Data Availability

The clinical imaging data analyzed in this study are not publicly available because they derive from retrospective patient imaging records and are subject to institutional and privacy restrictions. De-identified derived data may be available from the corresponding author upon reasonable request and subject to applicable institutional approval.

## Abbreviations

The following abbreviations are used in this manuscript: ALT, anterolateral thigh; AMT, anteromedial thigh; PMT, posteromedial thigh; PAP, profunda artery perforator; CTA, computed tomography angiography; LCFA, lateral circumflex femoral artery; PFA, profunda femoris artery; PACS, Picture Archiving and Communication System; SD, standard deviation.
